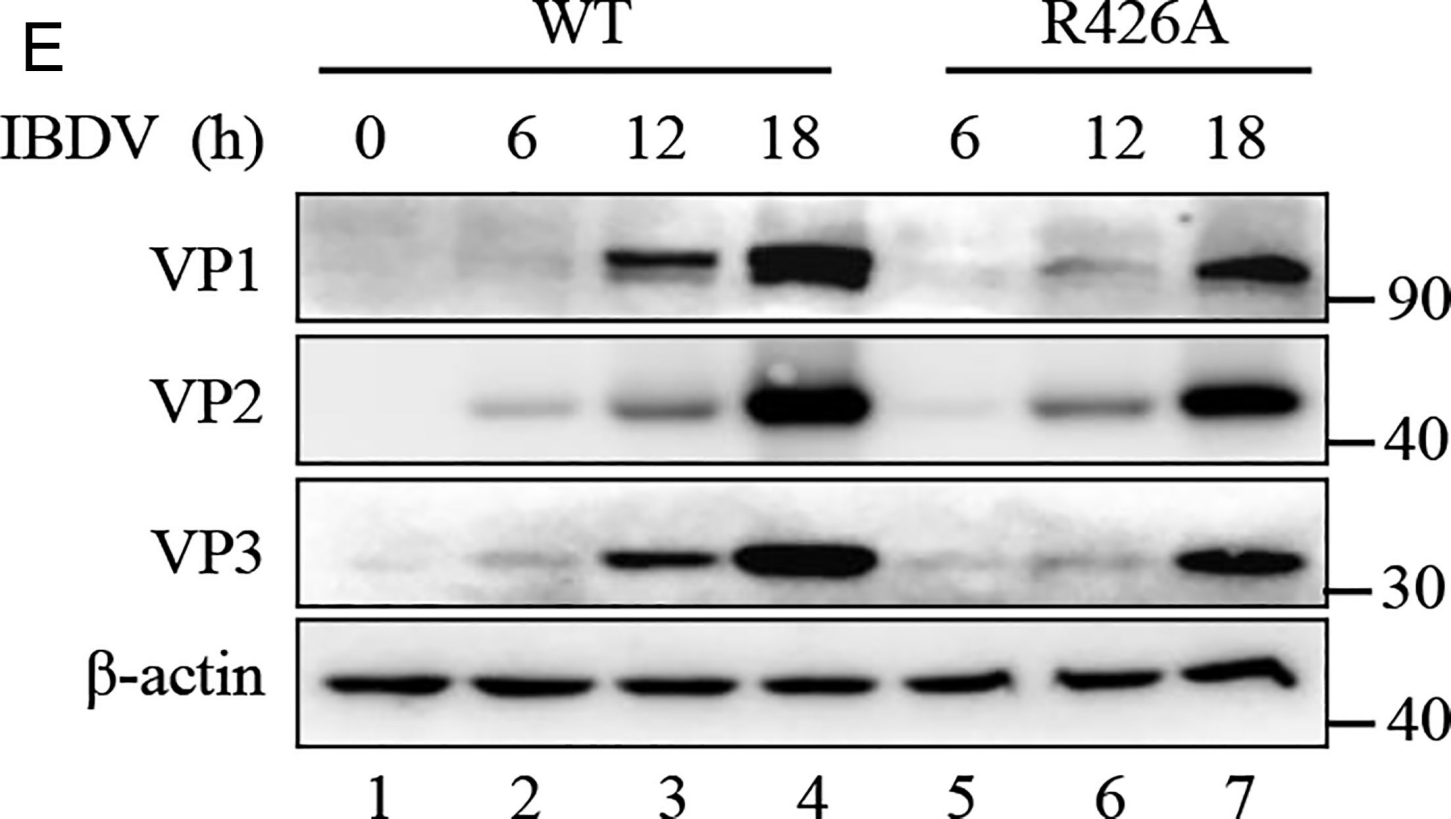# Correction for Hu et al., “PRMT5 Facilitates Infectious Bursal Disease Virus Replication through Arginine Methylation of VP1”

**DOI:** 10.1128/jvi.02002-25

**Published:** 2026-02-19

**Authors:** Xifeng Hu, Zheng Chen, Xiangdong Wu, Qiuling Fu, Zhen Chen, Yu Huang, Huansheng Wu

## AUTHOR CORRECTION

Volume 97, no. 3, e01637-22, 2023, https://doi.org/10.1128/jvi.01637-22. Figure 8E should appear as shown in this correction. After we carefully checked the original Western blot results, we found that, regretfully, we inadvertently used the wrong images in this panel. The current values are correct, and the conclusions remain intact. We apologize for this error, which did not change the final result that the growth rate of R426 mutant IBDV was significantly lower than that of wild-type (WT) IBDV.

**Fig 8:**